# Regulatory Mechanisms of Tannins on the Decomposition Rate of Mixed Leaf Litter in Submerged Environments

**DOI:** 10.3390/plants14193064

**Published:** 2025-10-03

**Authors:** Lisha Li, Jiahao Tan, Gairen Yang, Yu Huang, Yusong Deng, Yuhan Huang, Mingxia Yang, Jizhao Cao, Huili Wang

**Affiliations:** 1 Forestry College, Guangxi University, No. 100 Daxue Road, Nanning 530004, China; lishali2025@163.com (L.L.); tanjh63@mail2.sysu.edu.cn (J.T.); hymtmlsyya@163.com (Y.H.); denny2018@gxu.edu.cn (Y.D.); huangyuhan0710@foxmail.com (Y.H.); yangmx9495@163.com (M.Y.); 2 Guangxi Key Laboratory of Forest Ecology and Conservation, No. 100 Daxue Road, Nanning 530004, China; 3 Guangxi Laboratory of Forestry, No. 23 Yongwu Road, Nanning 530002, China; jizhaocao@163.com (J.C.); wanghuili6@163.com (H.W.)

**Keywords:** tannins, leaf litter decomposition rate, nitrogen cycle, bacterial community structure, water eutrophication

## Abstract

Terrestrial cross-boundary inputs of leaf litter serve as a critical foundation for secondary productivity in freshwater ecosystems. The regulatory mechanisms of tannins in leaf litter on degradation rates under submerged conditions remain unclear. This study employed leaf litter from low-tannin plants *Osmanthus fragrans* (A) and *Canna glauca* (B) as decomposition substrates, with the high-tannin species *Myriophyllum verticillatum* (C) incorporated to adjust tannin levels. A 140-day hydroponic degradation experiment was conducted under controlled temperature and dark conditions, which included four mixed litter treatments with a gradient of tannin additions (AB as the control, 0 g; ABC1: 0.5 g; ABC2: 2.5 g; ABC3: 4.5 g) along with two single-species treatments (A and B). The following results were found: (1) Low tannin levels (ABC1) promoted degradation rates of A and B (increased by 1.33–12.70%), whereas high tannin (ABC3) inhibited decomposition (decreased by 6.21–6.82%). (2) Tannin–protein complexes reduce nitrogen bioavailability and inhibit nitrification, thereby disrupting the nitrogen cycle in aquatic systems. In ABC3, total nitrogen content in A and B litter increased by 17.69–26.46% compared to AB, with concurrent 59.29% elevation in water NH_4_^+^-N concentration. (3) High tannin induced dominance of oligotrophic stress-resistant bacterial communities (e.g., *Treponema*) through nutrient limitation and toxicity stress; however, their low metabolic efficiency reduced overall decomposition efficiency. Research reveals that the ecological benefits of plant secondary metabolites outweigh their nutritional quality attributes.

## 1. Introduction

Freshwater ecosystems and their littoral zones form typical meta-ecosystems, tightly connected through cross-boundary flows of water, organic matter (e.g., litter), and organisms (e.g., insects) [1]. Terrestrially derived organic matter serves as a key basal resource supporting aquatic secondary production [2], yet its underlying mechanisms and relative contribution vary significantly across different freshwater systems. In stream ecosystems, the role of allochthonous leaf litter is relatively well-understood: it is rapidly processed through the detrital food web, thereby providing stable support for invertebrate production [3]. In contrast, the role of terrestrial organic matter in lake ecosystems is more complex. On one hand, terrestrial particulate organic carbon (POC) can be partially utilized via the benthic pathway [4]; however, in nutrient-rich lakes where internal primary production dominates, its contribution to overall secondary production is often limited [5]. On the other hand, terrestrial dissolved organic carbon (DOC) primarily stimulates bacterial production, but exhibits low transfer efficiency to higher trophic levels [6]. Although the role of terrestrial organic matter in lakes is known to be regulated by nutrient status and quality, a significant knowledge gap remains regarding the decomposition of terrestrially derived leaf litter (e.g., from riparian plants) in aquatic ecosystems, largely due to the historical separation of terrestrial and aquatic research [1]. The physicochemical properties of such litter inputs [7] differ markedly from those of aquatic plant litter. While organic matter decomposition in both environments is influenced by environmental conditions, litter traits, and decomposer organisms [8], terrestrial decomposition is typically slower [9], primarily due to lower moisture availability and inefficient invertebrate decomposers. Land–water material exchange occurs frequently, for example, litter shuttling reciprocally with water-level fluctuations or terrestrial litter entering aquatic systems, creating novel mixed-effects (e.g., high/low-tannin combinations) during in-water degradation. These exchange and decomposition processes significantly influence ecosystem functions (e.g., carbon/nitrogen cycling) in both realms, contingent on factors such as litter characteristics.

Leaf litter, a natural byproduct of plants, releases nutrients during its decomposition process, which are subsequently reabsorbed and utilized by plants, thereby influencing vegetation succession [10]. The primary determinant of the decomposition rate of leaf litter is its initial quality, which is governed by its intrinsic physical structure and chemical composition [7]. Leaf litter with relatively high initial quality generally contains elevated levels of N and P, which promote decomposition [11]. In contrast, low N content in litter can inhibit microbial growth and metabolic functions to varying degrees [12,13], slowing decomposition rates, as nitrogen is a critical element for microbial protein synthesis. Tannins are a class of phenolic polymers, classified as secondary metabolites produced by plants to adapt to their environment during growth and development [14]. They are widely distributed in plant leaves, bark, and fruits. The tannin content in plant leaves can be categorized into three levels: less than 20 mg/g indicates low-tannin plants, 20–50 mg/g indicates medium-tannin plants, and greater than 50 mg/g indicates high-tannin plants [11,15,16]. Tannins exhibit a significant phased role during the decomposition of leaf litter [17]. In the initial stage of litter decomposition, tannin content is at its highest, strongly inhibiting decomposition through mechanisms such as the formation of protein complexes and the suppression of enzyme activity. The inhibitory dynamics, however, are influenced by tannin type. Hydrolyzable tannins (HTs) leach rapidly upon immersion, creating an immediate pulse of inhibition in the surrounding water and on the litter surface. In contrast, condensed tannins (CTs) are often bound to cell wall matrices, leading to their slower release and more persistent suppression of microbial activity within the litter [17,18]. This combined action effectively locks in carbon and nitrogen, impeding nutrient release [19]. Concurrently, the leaching of soluble tannins further enhances the inhibitory effects on the remaining litter components. During the middle stage, as tannin concentrations decline, the microbial community undergoes restructuring. Tannin-tolerant taxa (e.g., certain fungi) become dominant [20], and their secreted enzymes gradually degrade the tannins. The nitrogen mineralization rate shows a trend of first decreasing and then increasing, and the decomposition process becomes more strongly regulated by environmental factors [11]. In the final stage, residual tannins form recalcitrant complexes with compounds like lignin, continuously inhibiting microbial and enzyme activity, which leads to a reduced litter decomposition rate [21]. This mechanism not only promotes the long-term sequestration of carbon in the form of humus but also significantly delays nitrogen mineralization, exerting a long-term influence on the ecosystem’s nitrogen cycle.

Some researchers have also reported that at low concentrations, tannins can serve as a carbon source for microorganisms, whereas at high concentrations, they may be toxic to microbial communities [7]. However, toxic tannin oligomers, formed from nontoxic tannin monomers, can be detoxified through further polymerization into higher-molecular-weight compounds [22]. These findings suggest that in environments with low concentrations of tannins, these compounds can act as substrates for microbial growth [23], promoting microbial proliferation and increasing leaf litter decomposition rates. High concentrations of tannins inhibit litter decomposition through multiple mechanisms. A primary mechanism is the complexation with proteins, which immobilizes nitrogen and impedes its microbial availability [19]. This process, however, varies considerably between the two main tannin classes. CTs, as larger polymeric flavonoids, form stable, often insoluble complexes with proteins via multiple hydrogen bonds and hydrophobic interactions [16,18]. In contrast, HTs, being smaller ester-based molecules, exhibit lower binding affinity and a reduced tendency to precipitate proteins [18]. Beyond nitrogen immobilization, tannins can directly suppress decomposition by binding to key extracellular enzymes, such as cellulase and lignin peroxidase. This interaction can occlude active sites or alter enzyme conformation, thereby diminishing catalytic activity [24]. Simultaneously, tannins complex organic nitrogen and metal ions via their phenolic hydroxyl groups, reducing the nitrogen mineralization rate and limiting the nitrogen supply for microbes [25]. At the microbial level, high-tannin environments suppress the growth of most bacteria, promoting tannin-tolerant fungi and certain bacteria to become dominant groups [20,26]. This leads to a decrease in microbial diversity and an imbalance in functional community structure, manifested as a reduced abundance of cellulose- and lignin-degrading microorganisms [17]. Additionally, tannin–protein complexes increase the mechanical strength and structural density of litter, further delaying microbial colonization and physical fragmentation [19]. Collectively, these processes result in a significant reduction in the release rates of carbon and nitrogen, promote the long-term stable retention of organic carbon, and inhibit the ecosystem nitrogen cycle. Extended periods are required for the complete degradation of recalcitrant compounds in leaf litter, such as tannins and lignin [11], and the regulatory effects of these recalcitrant substances on leaf litter decomposition remain insufficiently studied.

In natural ecosystems, plant leaf litter typically decomposes in mixed forms, and the physical and chemical compositions of litter from different species vary significantly. These differences can influence decomposition rates, with mixed litter decomposition exhibiting both additive and nonadditive effects [27]. These effects are determined by comparing the actual decomposition rates of mixed litter with the rates predicted on the basis of single-species litter decomposition. Additive effects occur when the decomposition rate of mixed litter is equivalent to the average decomposition rate of individual species, whereas nonadditive effects include both synergistic and antagonistic effects [28]. Synergistic effects are considered to occur when mixed litter decomposes faster than the rate predicted from single-species litter decomposition, whereas antagonistic effects are considered to occur when the decomposition rate of mixed species litter is slower than that predicted from single-species litter decomposition [29]. Typically, the microbial abundance and diversity in mixed litter are significantly greater than those in single-species litter [30]. Mixed litter provides diversified carbon and nitrogen sources for microorganisms, enabling the complementary utilization of these resources [13,31] and fostering diverse microbial metabolic pathways and increased decomposition activity. Interactions among the decomposition environment, litter substrate, and dominant decomposers at different decomposition stages [32] can either promote or inhibit mixed litter decomposition [33]. However, there is no consensus on how the diversity, abundance, and breadth of litter from different species influence mixed litter decomposition rates. Therefore, a deeper understanding of the dominant factors affecting decomposition rates in mixed litter and further exploration of the mechanistic roles of unconventional elements in mixed litter decomposition are needed.

Previous studies on terrestrial ecosystems have revealed that tannins play a critical role in nutrient cycling, particularly in nitrogen cycling, during leaf litter decomposition [34]. For example, at high concentrations, tannins can inhibit soil nitrogen mineralization [19], primarily by reducing microbial activity and soil enzyme activity [7], thereby affecting nitrogen mineralization processes. Tannins can complex with proteins and various nonprotein organic nitrogen compounds [35]. The multiple phenolic hydroxyl groups in tannin structures can form hydrogen bonds with the amide groups of proteins, resulting in stable complexes [36]. The factors influencing tannin–protein complexation are diverse [36] and include tannin structure and concentration, protein type and concentration, and pH [35]. While the mechanisms of tannin-mediated soil nutrient cycling in terrestrial ecosystems are becoming clearer, few studies have explored the effects of tannins on leaf litter decomposition in aquatic environments. Generally, prolonged immersion and physical abrasion in water accelerate the fragmentation of leaf litter [2], releasing substantial nutrients that can provide favorable conditions for microbial growth and proliferation. Diverse microbial communities facilitate efficient litter degradation [30,32], and changes in water physicochemical properties can significantly influence decomposer activity. These alterations not only affect leaf litter decomposition rates but may also have profound implications for nutrient cycling in aquatic ecosystems. Terrestrial plant litter often enters aquatic systems and coexists with aquatic plant litter during decomposition. The continuous accumulation of released nutrients may contribute to water eutrophication, highlighting the necessity of understanding the long-term impacts of the decomposition of mixed plant litter on aquatic environments.

Therefore, we selected three plants with significantly different tannin contents that are commonly used in the riparian zone of urban water bodies, namely, *Osmanthus fragrans*, *Canna glauca*, and *Myriophyllum verticillatum*, and established various experimental decomposition groups. In this study, we aimed to address the following key questions: (1) How do tannins regulate aquatic leaf litter decomposition rates? (2) What are the characteristics of nitrogen dynamics during aquatic litter decomposition, and how do they influence water quality? (3) What are the dominant factors driving changes in bacterial community structure and function, and how do these changes correlate with aquatic litter decomposition rates and nutrient release? By addressing these questions, we aim to deepen the understanding of leaf litter decomposition associated with tannins and explore litter mixtures of both terrestrial and aquatic plant litters.

## 2. Results and Analysis

### 2.1. Characteristics of Leaf Litter Decomposition Rates

**Figure 1:**
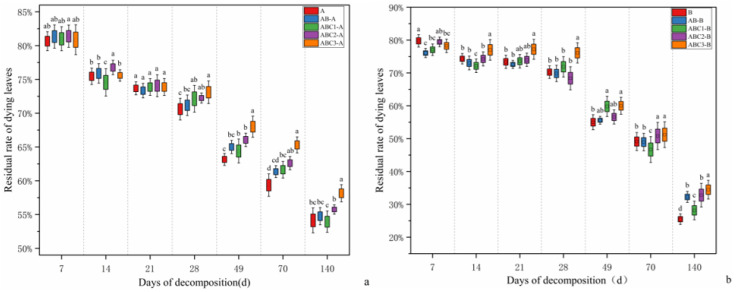
(**a**,**b**) show the leaf litter retention rates of *Osmanthus fragrans* and *Canna glauca*, respectively. The figure shows differences in litter residual rates among different treatment groups during decomposition (7, 14, 21, 28, 49, 70, and 140 days). Values are presented as mean ± SE (*n* = 3), with different lowercase letters in the figure indicating significant differences between groups at the *p* < 0.05 level (determined by Tukey’s test). This allows for a visual comparison of changes in residual rates across different decomposition stages and differences amongst treatment groups.

As shown in Figure 1, significant differences were observed in the decomposition rates of *Osmanthus fragrans* and *Canna glauca* leaf litters among the different treatments.

#### 2.1.1. Decomposition of *Osmanthus fragrans*

On day 14 of decomposition, the rate of residual *Osmanthus fragrans* in the ABC1-A treatment group was significantly lower than that in all other treatment groups. By day 21, no significant differences were detected in the rates of residual *Osmanthus fragrans* among the treatments, with residual rates ranging between 73.00% and 74.00%. On day 28, the rate of residual *Osmanthus fragrans* in the ABC3 treatment group significantly exceeded that in the other four treatment groups. From day 49 to day 140, the rates of residual *Osmanthus fragrans* followed the order ABC3-A > ABC2-A > AB-A > ABC1-A > A. At the end of the experiment, the residual rates in each treatment were A (54.00%), AB-A (54.75%), ABC1-A (53.80%), ABC2-A (55.15%), and ABC3-A (58.75%).

#### 2.1.2. Decomposition of *Canna glauca*

On day 7 of decomposition, the residual rate of residual *Canna glauca* in treatment B was significantly greater than that in all other treatments. By day 14, the rates of residual *Canna glauca* across treatments ranged between 73.00% and 77.00%, with the residual rate in treatment ABC1-B being significantly lower than that in the other treatment groups.

From day 21 to day 140, the residual rate in the ABC3-B treatment remained significantly greater than that in all the other treatments. On day 70, the residual percentage in the ABC1-B treatment was the lowest at 46.65%. In the later stages of the experiment, the decomposition rate of *Canna glauca* litter was faster than that of *Osmanthus fragrans*. At the end of the experiment (day 140), the residual rates for each treatment were as follows: B (25.60%), AB-B (32.25%), ABC1-B (28.15%), ABC2-B (32.80%), and ABC3-B (34.45%).

#### 2.1.3. Decomposition Characteristics of Mixed Leaf Litter

The residual rate of the *Myriophyllum verticillatum* single-species litter degradation group (Treatment C) was 38.00% on day 140. Using Equation (2) from Section 4.5, the theoretically predicted residual rates for the mixed degradation groups (AB, ABC1, ABC2, and ABC3) were calculated as 39.80%, 39.51%, 38.92%, and 38.66%, respectively. However, the actual residual rates were 43.50%, 39.22%, 41.17%, and 41.96%, respectively. These results indicate that in this study, the decomposition of mixed aquatic and terrestrial leaf litter had nonadditive effects. Specifically, treatment ABC1 had a synergistic effect, as its actual residual rate was lower than the predicted value. Treatments AB, ABC2, and ABC3 exhibited antagonistic effects, as their actual residual rates were higher than the predicted values.

Using Equation (3) from Section 4.5, the decomposition rates (*k*) for each treatment group were calculated (Table 1). The key findings included the following: for *Osmanthus fragrans* and *Canna glauca* litters in Treatment ABC3, the *t*_0.95_ values were significantly greater than those in the other treatments at 2158.49 days and 771.12 days, respectively. The single-species litter degradation groups (A and B) presented the lowest *t*_0.95_ values, at 1521.19 days and 616.72 days, respectively. The *t*_0.95_ values for *Canna glauca* litter in treatments AB and ABC2-B were similar, at 741.95 days and 741.68 days, respectively. Across all treatments, the *t*_0.95_ values followed the order ABC3 > ABC2 > AB > ABC1 > A/B, which aligns with the trends observed in the residual rates.

The results indicated that in the single-species degradation groups (A and B), *Osmanthus fragrans* and *Canna glauca* litters had the fastest decomposition rates. Compared with the mixed degradation group without tannin addition (AB), the mixed degradation group with 0.50 g added tannins (ABC1) presented a faster decomposition rate. The degradation rate of litter in the ABC2 group (with 2.50 g added tannins) was similar to that of the AB group but slightly slower. Compared with the other treatment groups, the ABC3 group (supplemented with 4.50 g tannins) presented a significantly lower decomposition rate. These findings demonstrate that tannin addition has a low-promotion and high-inhibition effect on the decomposition rates of mixed leaf litter substrates. Specifically, at low-tannin addition (e.g., 0.50 g), the decomposition rate of mixed leaf litter was accelerated. A high level of tannin addition (e.g., 4.50 g) significantly lowered the decomposition rate, indicating that decomposition is inhibited when a critical tannin threshold is exceeded.

### 2.2. Changes in Tannin Content During Leaf Litter Decomposition

**Figure 2:**
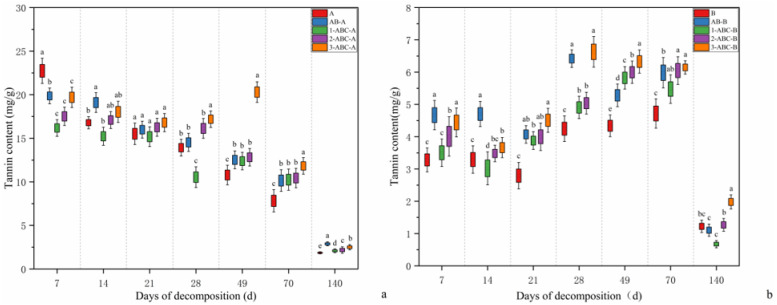
(**a**,**b**) are box plots showing the changes in tannin content during the decomposition process of fallen leaves of *Osmanthus fragrans* and *Canna glauca* in different treatment groups, respectively. Data in both figures are presented as mean ± standard error (*n* = 3). Box plots display the distribution characteristics of the data (including median, quartiles, outliers, etc.). Different lowercase letters above the boxes indicate significant differences amongst groups at the *p* < 0.05 level, which can be used to compare the dynamic changes in tannin content over decomposition time and the pattern of differences amongst treatment groups.

As shown in Figure 2, the tannin content in *Osmanthus fragrans* leaf litter exhibited the following trends. Initial phase (0–7 days): The tannin content increased in treatments A, AB, and ABC3 but decreased in the other treatment groups. In treatment A, the tannin content was significantly greater than that in the other groups, while that in the three tannin-treated groups (ABC1, ABC2, and ABC3) was not significantly different. Mid-phase (14–70 days): The tannin content in all the treatment groups began to decrease from day 14 onward. The tannin content in treatment ABC3 remained significantly higher than that in the other four treatment groups from day 14 to day 70. Later phase (70–140 days): The tannin content in treatment ABC3 decreased most rapidly, while that in treatment A showed the smallest decline. Final tannin content (day 140): The tannin content in treatments A, AB-A, ABC1-A, ABC2-A, and ABC3-A was 1.85, 2.88, 2.09, 2.18, and 2.50 mg/g, respectively. The overall reduction in tannin content compared with the initial level was as follows: A (90.08%), AB-A (84.57%), ABC1-A (88.83%), ABC2-A (88.35%), and ABC3-A (86.64%).

Initial phase (0–7 days): The tannin content in *Canna glauca* leaf litter decreased rapidly in all the treatment groups. The reduction rates were as follows: B (73.11%), AB-B (61.74%), ABC1-B (71.29%), ABC2-B (67.14%), and ABC3-B (63.60%). Days 14–28: The tannin content in treatments B and ABC3 initially decreased but then increased. By day 28, the tannin content in the ABC3 and AB treatments was significantly greater than that in the other groups. Days 28–140: The tannin content in all five treatment groups decreased rapidly. At the end of the experiment (day 140), the tannin content in the ABC3 treatment group remained significantly higher than that in the other groups. Final tannin content (day 140): The overall reduction in tannin content compared with the initial level was as follows: B (89.97%), AB-B (90.95%), ABC1-B (94.49%), ABC2-B (89.60%), and ABC3-B (83.78%). The greatest reduction occurred in the ABC1 treatment, whereas the smallest reduction was observed in the ABC3 treatment.

### 2.3. Changes in Total Nitrogen (TN) Content and the Nitrogen Accumulation Index (NAI) During Leaf Litter Decomposition

#### 2.3.1. *Osmanthus Fragrans* TN Content and NAI Dynamics

**Figure 3:**
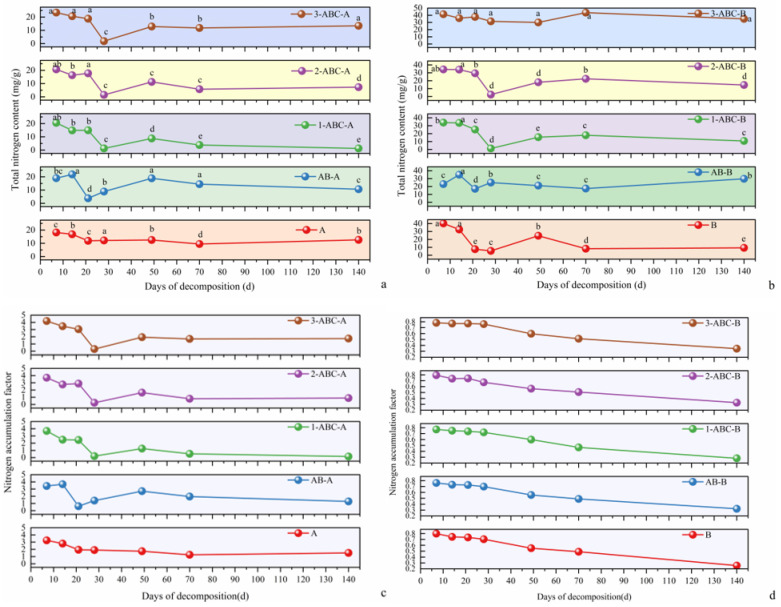
(**a**,**b**) show the changes in total nitrogen content of the leaf litter of *Osmanthus fragrans* and *Canna glauca* under different treatment groups, respectively. (**c**,**d**) on the other hand, illustrate the variation trends of the nitrogen accumulation coefficient of the leaf litter of *Osmanthus fragrans* and *Canna glauca* during the decomposition period (0–140 days), respectively. Data are presented as mean ± SE (*n* = 3). The lines and data points illustrate the dynamic changes in total nitrogen content and nitrogen accumulation factor over decomposition time for each treatment group. Different lowercase letters next to the data points indicate significant differences amongst groups at the *p* < 0.05 level, which can be used to analyze the patterns of changes in TN content and the inter-group differences in nitrogen accumulation or release during decomposition.

Changes in the total nitrogen (TN) content in osmanthus leaf litter across the various decomposition treatment groups during the degradation process are shown in Figure 3. Days 0–7: The TN content in all the treatment groups increased sharply. The NAI values also increased rapidly, indicating a net accumulation of nitrogen during this phase. Days 21–28: The TN content in the three tannin-treated groups (ABC1, ABC2, and ABC3) decreased rapidly. The Net Nitrogen Accumulation Index (NAI-N) values for these groups decreased sharply, transitioning from values greater than 1 to values less than 1. By day 28, the TN content in treatments A and AB was significantly greater than that in the tannin-supplemented groups, reaching values of 12.18 mg/g and 8.90 mg/g, respectively. Days 28–70: The TN content in all the treatment groups initially increased but then decreased. The final TN content and NAI (day 140) in treatments A, AB-A, ABC1-A, ABC2-A, and ABC3-A were 12.64, 10.65, 1.28, 7.30, and 13.47 mg/g, respectively. The NAI-N values were less than 1 for treatments ABC1 and ABC2, indicating nitrogen release, whereas the values were greater than 1 for treatments A, AB, and ABC3, indicating nitrogen accumulation.

#### 2.3.2. *Canna glauca* TN Content and NAI Dynamics

General Trends: Except treatments B and ABC1-B, which presented an overall decline in TN content, the treatment groups presented an increasing trend in TN content. Throughout the decomposition period, the NAI-N values for all the treatment groups were less than 1, indicating net nitrogen release. Days 0–7: The TN content in all the treatment groups increased sharply. Despite rapid mass loss during this phase, the NAI-N values remained less than 1, indicating net nitrogen release. Days 14–28: The TN content in treatments B, ABC1-B, and ABC2-B decreased sharply, reaching the lowest levels over the entire decomposition period. After day 28, the TN content in these groups increased rapidly. Days 28–140: The TN content in the ABC1-B and ABC2-B treatments decreased, whereas it increased in the other treatment groups. The final TN content and NAI (day 140) values for the tannin-treated groups were as follows: ABC3-B: 35.08 mg/g (34.45%), ABC2-B: 14.63 mg/g (32.28%), and ABC1-B: 10.78 mg/g (28.15%).

### 2.4. Impact of Leaf Litter Decomposition on Water Quality

#### 2.4.1. Changes in Tannin and TN Content in Water

**Figure 4:**
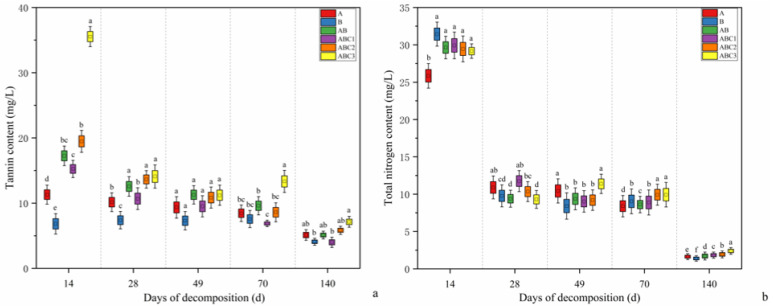
(**a**,**b**) show box plots of the tannin and total nitrogen contents in water under different treatment groups during the leaf litter decomposition process (on days 14, 28, 49, 70, and 140), respectively. Data are presented as mean ± SE (*n* = 3), with different lowercase letters indicating significant differences amongst groups at the *p* < 0.05 level (determined by Tukey’s test).

Changes in the tannin content in the water across the different treatment groups during the 140-day decomposition period are shown in Figure 4. At the beginning of the experiment, the tannin content in the water was 2.51 mg/L. During the early stages of decomposition, the tannin content in the water increased sharply but then decreased in all the treatment groups. Specifically, during the initial phase (0–14 days), the tannin content in the water increased rapidly in all the treatment groups over the first 14 days. Except treatment B, the treatment groups presented peak tannin contents in the water on day 14. In treatment B, the peak tannin content was reached on day 28. Later phase (70–140 days): The tannin content in the water tended to decrease in all the treatment groups. By the end of the experiment, the ABC3 treatment group presented a significantly greater tannin content in the water than the other groups did, and the tannin content of the ABC3 treatment group remained higher than that of the other treatment groups throughout the experiment. On day 140, the tannin content in the water increased by 103.32%, 62.67%, 103.36%, 58.98%, 132.25%, and 183.81% compared with the initial levels in the respective treatment groups.

The initial TN content in the water was 1.26 mg/L. During the experiment, the TN content in the water across all the treatment groups rapidly increased, followed by a decrease over the 140-day decomposition period. The key observations included the following: Days 0–7: The TN content in the water increased sharply in all the treatment groups within the first 7 days. In all the treatment groups, the peak TN content in the water was reached on day 14, with that in treatment A being significantly lower than those in the other five groups. Mid-phase (14–28 days): The TN content in the water decreased rapidly in all the treatment groups. By day 28, the ABC1 treatment group had a significantly greater TN content in the water than did the other groups. Later phase (28–140 days): From days 28 to 49, the TN content in the water increased in treatment ABC3 but decreased in the other five treatment groups. From days 49 to 140, the ABC3 treatment consistently resulted in a significantly greater TN content in the water than did the other treatments. During the first 70 days, the TN content in the water generally increased across all the treatment groups, but it decreased significantly in the later stages of the experiment.

#### 2.4.2. Changes in the NH_4_^+^-N and NO_3_^−^-N Contents in Water

**Figure 5:**
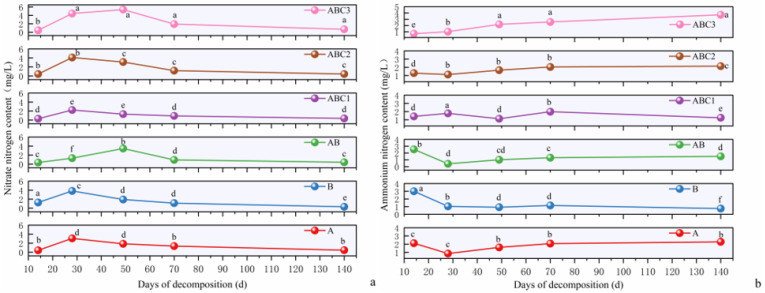
(**a**,**b**) illustrate the variation trends of ammonium nitrogen and nitrate nitrogen contents in water under different treatment groups during the leaf litter decomposition process, respectively. Data are presented as mean ± SE (*n* = 3), with different lowercase letters indicating significant differences amongst groups at the *p* < 0.05 level (determined by Tukey’s test).

The changes in the NH_4_^+^-N content in the water across the different treatment groups during the 140-day decomposition period are shown in Figure 5. At the beginning of the experiment, the NH_4_^+^-N content in the water was 1.27 mg/L. The key observations included the following: Initial phase (0–14 days): Except in the ABC3 treatment, the NH_4_^+^-N content in the water increased in the treatment groups. Treatment B resulted in the most significant increase, resulting in the highest NH_4_^+^-N content during the experiment. Mid-phase (28–49 days): The NH_4_^+^-N content decreased in treatments B and ABC1 but increased in the other four treatment groups. By day 49, the ABC3 treatment group presented a significantly greater NH_4_^+^-N content than the other groups did. Later phase (49–140 days): The NH_4_^+^-N content decreased in treatments B and ABC1 but increased in treatments A, AB, ABC2, and ABC3, with treatment ABC3 showing the most significant increase. At the end of the experiment, the NH_4_^+^-N contents in the water for treatments A, B, AB, ABC1, ABC2, and ABC3 were 2.28, 0.75, 1.51, 1.25, 2.15, and 3.72 mg/L, respectively.

The initial NO_3_^−^-N content in the water was 0.49 mg/L. The key observations included the following: Initial phase: Except in treatment B, the NO_3_^−^-N content in the water decreased in the treatment groups, reaching the lowest levels in the experiment. 14–28 days: The NO_3_^−^-N content increased rapidly in all the treatment groups. In treatments A, B, ABC1, and ABC2, the NO_3_^−^-N content peaked on day 28. Days 28–49: The NO_3_^−^-N content increased in the AB and ABC3 treatments, reaching its highest level during the decomposition period, whereas it decreased in the other four treatment groups. By day 49, the ABC3 treatment group presented a significantly greater NO_3_^−^-N content than the other groups did. Final phase (49–140 days): The NO_3_^−^-N content in the water continued to decrease in all the treatment groups.

### 2.5. Bacterial Community Changes During Different Stages of Leaf Litter Decomposition Changes in Bacterial Diversity

During the first 30 days of decomposition, the bacterial diversity and richness of *Osmanthus fragrans* leaf litter increased with increasing tannin concentration across all treatment groups (Table S1). In the mid-decomposition stage, the Simpson index for *Osmanthus fragrans* leaf litter decreased, whereas the Shannon index increased, with the highest values observed in the low-tannin treatment groups. Similarly, the Chao1 and ACE indices were highest in the low-tannin treatment groups.

For *Canna glauca* leaf litter (Table S2), the Simpson and Shannon indices in treatments B, AB, ABC1, ABC2, and ABC3 generally decreased compared with those in the initial stage, with no significant differences among the three tannin-supplemented groups. The Chao1 and ACE indices were highest in treatment B2 and lowest in treatment AB2-B.

In the later stages of decomposition, the Chao1 and ACE indices for *Osmanthus fragrans* leaf litter were highest in the L3.ABC1.A. For *Canna glauca* leaf litter, the Shannon index peaked at 5.48 in treatment B3, while the Chao1 and ACE indices increased rapidly, with the highest values similarly occurring in treatment B3. Among the tannin-treated samples, the highest values were observed in treatment L3.ABC1.B.

#### 2.5.1. Bacterial Community Structure at the Phylum Level

**Figure 6:**
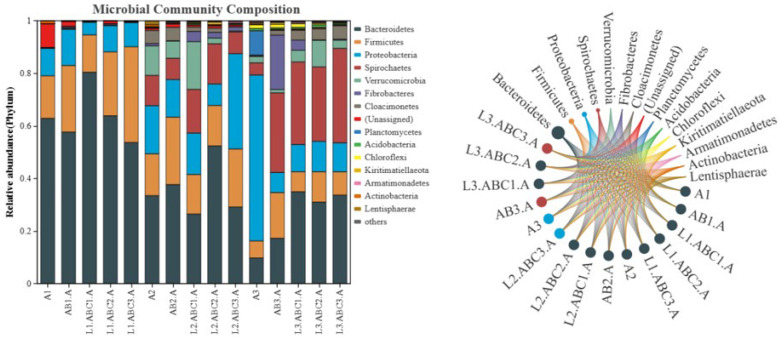
Bacterial community structure at the phylum level in *Osmanthus fragrans* litter across different treatment groups. Taxa with a relative abundance of ≥0.01% were selected at the phylum level, and the top 15 taxa by abundance are shown to illustrate the bacterial community composition. The chord diagram reflects the composition ratio of dominant species in each sample and simultaneously displays the distribution ratio of each dominant species across different samples.

As shown in Figure 6, the dominant bacterial phyla in the *Osmanthus fragrans* leaf litter during the 140-day decomposition period included *Bacteroidetes*, *Firmicutes*, *Proteobacteria*, *Spirochaetes*, *Verrucomicrobia*, and *Fibrobacteres*. *Bacteroidetes* was the top phylum throughout the decomposition process. In the early stages, the abundance of *Bacteroidetes* was highest in the low-tannin treatment groups and lowest in the high-tannin groups.

In the mid-decomposition stage, *Bacteroidetes* remained the dominant phylum, but its relative abundance decreased significantly, while the relative abundances of *Proteobacteria*, *Spirochaetes*, and *Verrucomicrobia* increased. On day 140, the dominant phyla and their relative abundances in each treatment group were as follows: A3: *Proteobacteria* (63.13%), AB3-A: *Spirochaetes* (30.28%), L1.ABC1.A: *Bacteroidetes* (34.96%), L1.ABC2.A: *Bacteroidetes* (31.01%), L1.ABC3.A: *Spirochaetes* (35.95%).

**Figure 7:**
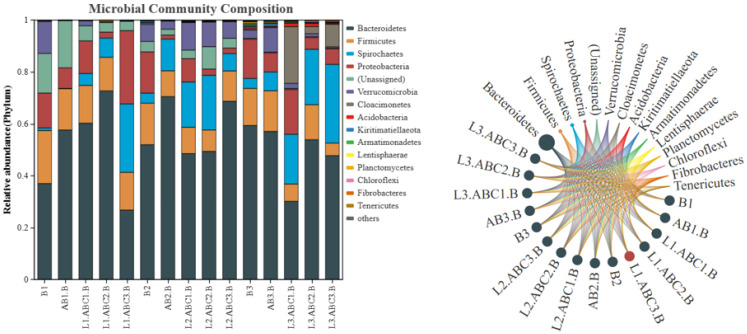
Bacterial community structure at the phylum level in *Canna glauca* litter across different treatment groups. Bacterial community structure at the phylum level in *Canna glauca* litter across different decomposition stages and treatment groups. Among them, *Bacteroidetes*, *Firmicutes*, *Spirochaetes*, and *Proteobacteria* are the dominant phyla.

As shown in Figure 7, the dominant bacterial phyla in *Canna glauca* leaf litter across the different treatment groups during the experimental period included *Bacteroidetes*, *Firmicutes*, *Spirochaetes*, *Proteobacteria*, and *Verrucomicrobia*. On day 30 of decomposition, *Bacteroidetes* was the dominant phylum in all the treatment groups except L1.ABC3.B, where *Proteobacteria* dominated (28.23%). The relative abundance of Bacteroidetes varied significantly among the different treatment groups. During the mid-decomposition stage of *Canna glauca* leaf litter, *Bacteroidetes* remained the dominant phylum, and its relative abundance increased compared with that in the initial stage. The relative abundance of *Bacteroidetes* in each treatment group decreased in the following order: AB2-B (70.52%) > L2.ABC3. B (68.69%) > B2 (51.98%) > L2.ABC2.B (49.41%) > L1.ABC2.B (48.52%). By day 140 of decomposition, *Bacteroidetes* remained the dominant phylum in all the treatment groups. During this stage, the relative abundances of *Firmicutes*, *Spirochaetes*, and *Proteobacteria* tended to increase.

#### 2.5.2. Analysis of Bacterial Community Structure at the Genus Level

**Figure 8:**
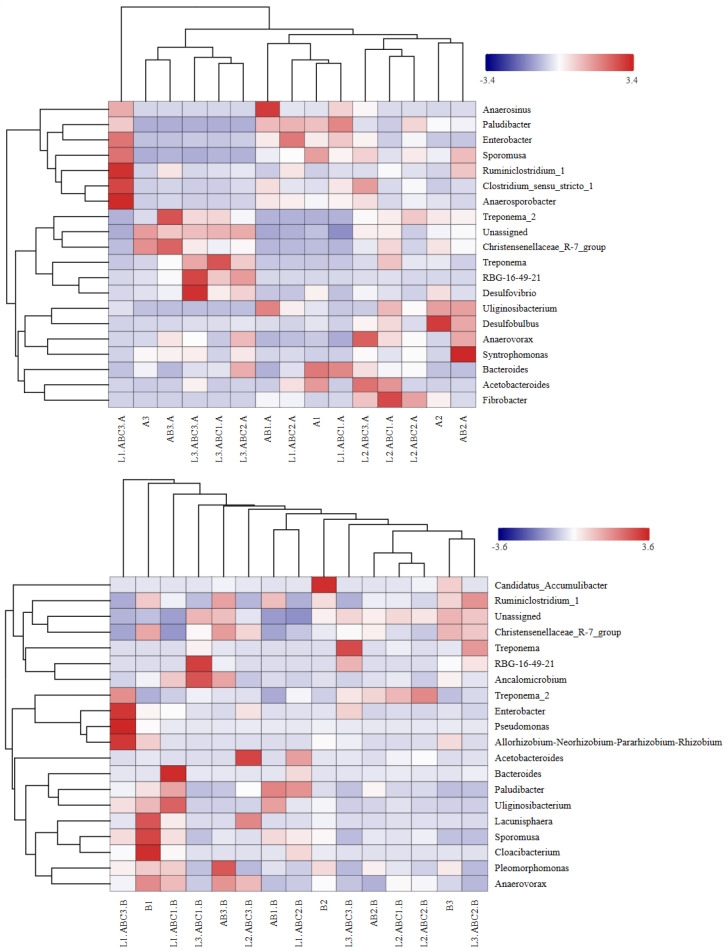
Species richness-based clustering analysis of genus-level bacterial communities from two types of fallen leaves. This diagram focuses on the top 20 species by abundance: the horizontal axis represents samples, and the vertical axis represents species. The clustering tree on the left is the species clustering tree, while the clustering tree at the top is the sample clustering tree, which reflects the similarity of community composition among samples. The values corresponding to the middle squares indicate the relative abundance of each row of species; the redder the square color, the higher the abundance of the species. It should be noted that this diagram only allows horizontal comparison (abundance differences in the same species across different samples) and not vertical comparison (abundance differences between different species). “-” represents a negative sign; the same applies below.

A further analysis of the community composition at the genus level (Figure 8) revealed that during the 140-day degradation period of *Osmanthus fragrans* leaf litter, the predominant bacterial genera were *Paludibacter*, *Treponema_2*, *Uliginosibacterium*, *Ruminiclostridium_1*, *Acetobacteroides*, *Treponema*, and *Enterobacter*. *Paludibacter*, classified as a member of *γ-proteobacteria*, was the top genus; it plays a critical role in carbon and nitrogen cycling, as well as in the degradation of organic matter. On day 30 of litter degradation, the abundance of *Paludibacter* significantly increased in the low-level tannin treatment, whereas its abundance increased with increasing tannin concentration. During the mid-degradation phase, *Paludibacter* remained the predominant genus across all the degradation groups; however, in contrast to its abundance in the early degradation phase, its relative abundance decreased. By day 140, *Treponema_2* emerged as the predominant genus across all the degradation groups. In the three low-tannin treatment groups, *Treponema* was the second most abundant genus across all groups, with the highest relative abundance observed in the low-tannin degradation group.

During the 140-day degradation period of *Canna glauca* leaf litter, the dominant bacterial genera at the genus level were *Paludibacter*, *Treponema_2*, *Ruminiclostridium_1*, *Acetobacteroides*, *Uliginosibacterium*, *Treponema*, *Christensenellaceae_R-7_group*, and *Lacunisphaera*, among others. In the early stages of *Canna glauca* leaf litter degradation, except in the L1.ABC3.B group, where the dominant genus was *Treponema_2* (18.19%), *Paludibacter* was the dominant genus in the groups. During the mid-degradation stage, *Paludibacter* remained the dominant genus in all the groups except L1.ABC3.B, but its relative abundance decreased in all the groups. By day 140 of degradation, the dominant genera and their relative abundances in each degradation group were as follows: B3 group: *Ruminiclostridium_1* (8.45%), AB3.B group: *Ruminiclostridium_1* (6.13%), L3.ABC1.B group: *Treponema_2* (6.02%), L3.ABC2.B group: *Treponema* (6.71%), and L3.ABC3.B group: *Treponema* (10.77%).

#### 2.5.3. Correlations of Bacterial Community Structure with Leaf Litter Decomposition Rate and Tannin Content

**Figure 9:**
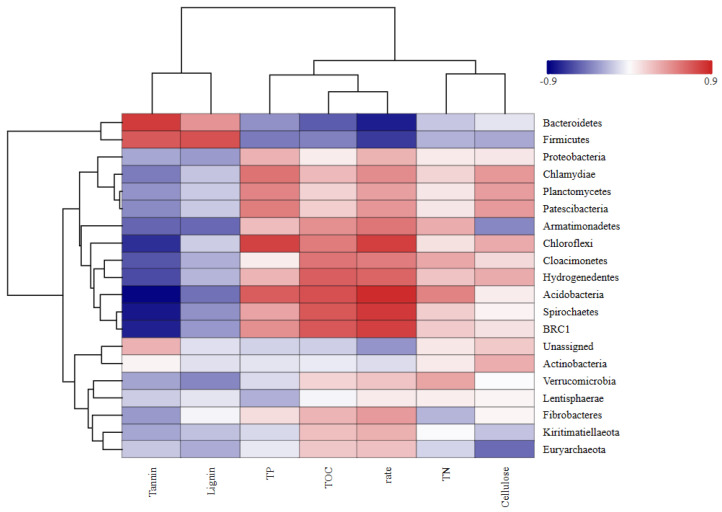
Correlation analysis of withered leaves of *Osmanthus fragrans* with degradation rate and physicochemical factors. Pearson correlation analysis between the top 10 phyla (by bacterial population richness) and litter decomposition rate, as well as contents of tannin, TN, TP, TOC, cellulose, and lignin during decomposition of *Osmanthus fragrans* litter. In the figure, the horizontal axis represents species (i.e., the above-mentioned top 10 phyla), and the vertical axis represents phenotypic indicators of samples (decomposition rate and contents of various substances). Blue indicates a negative correlation, and red indicates a positive correlation; the darker the color, the stronger the correlation.

**Figure 10:**
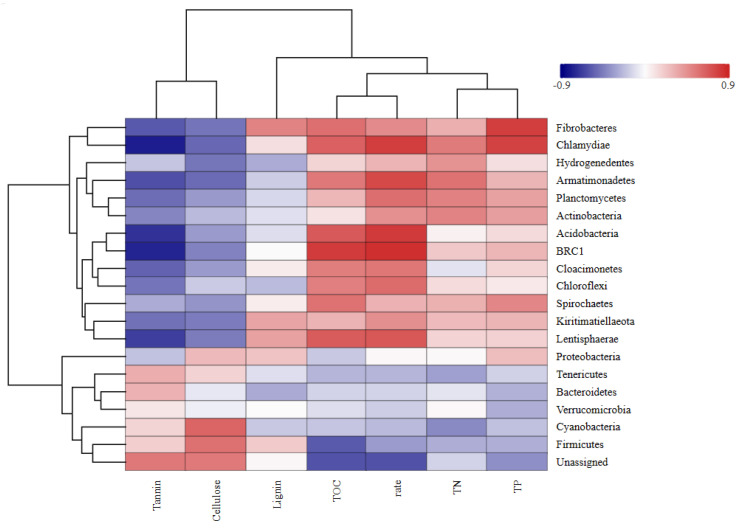
Correlation analysis of withered leaves of *Canna glauca* with degradation rate and physicochemical factors.

The results are illustrated in Figure 9 and Figure 10. During the degradation of *Osmanthus fragrans* leaf litter, significant correlations were observed for *Bacteroidetes* and *Firmicutes*. Both phyla exhibited a significant positive correlation with tannin content and a significant negative correlation with the decomposition rate. *Spirochaetes* and *Chloroflexi* were significantly negatively correlated with the tannin content but positively correlated with the decomposition rate. The phylum *Actinobacteria* was significantly positively correlated with total organic carbon (TOC). *Chloroflexi* was significantly positively correlated with TP. *Firmicutes* was significantly positively correlated with lignin content. During the degradation process of *Osmanthus fragrans* leaf litter, *Bacteroidetes* was positively correlated with tannins, cellulose, and lignin but negatively correlated with TOC, total Carbon(TC), and TN. In contrast, *Firmicutes* was negatively correlated with TOC, TC, TN, and cellulose.

In the degradation process of *Canna glauca* leaf litter, *Actinobacteria* and *Chlamydiae* were significantly negatively correlated with tannin content but positively correlated with the decomposition rate. *Fibrobacteres* and *Chlamydiae* exhibited significant positive correlations with total phosphorus (TP). *Firmicutes* was significantly negatively correlated with TOC.

**Figure 11:**
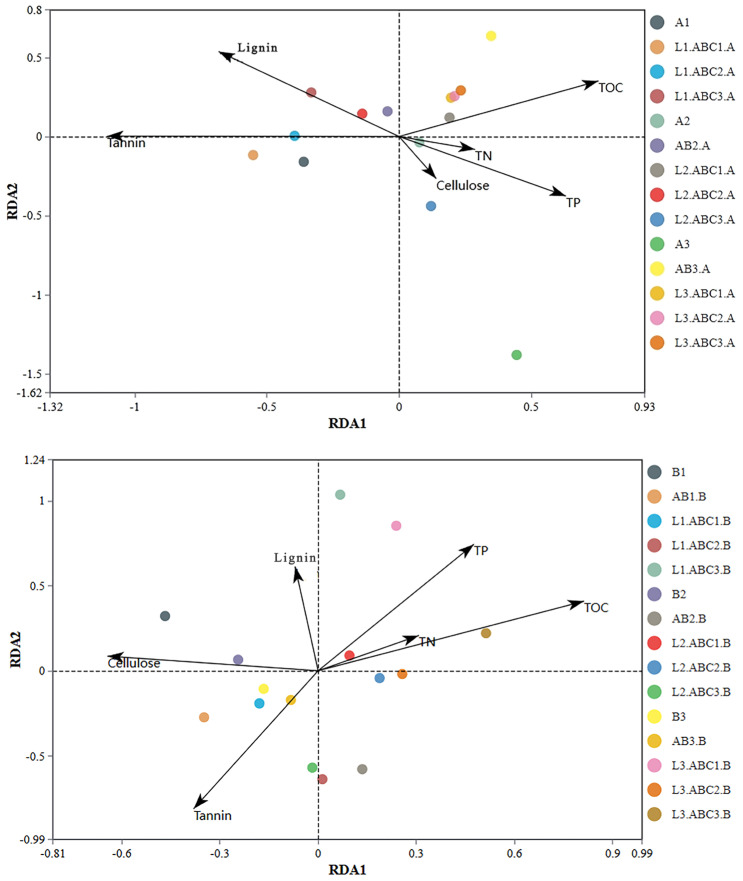
Chemical properties of fallen leaves of *Osmanthus fragrans* and *Canna glauca* and redundancy analysis of bacteria. In the figure, arrows represent different environmental factors (i.e., contents of the above substances), and different points represent samples. The angle amongst arrows reflects the correlation between environmental factors: an acute angle indicates a positive correlation, while an obtuse angle indicates a negative correlation. The length of the arrows represents the degree of influence.

Redundancy analysis was employed to investigate the impact of the physicochemical properties of the leaf litter on bacterial communities. The results indicated that during the experimental period for *Osmanthus fragrans* leaf litter, the first and second principal component axes explained 81.00% and 12.90% of the variation in bacterial communities, respectively. As shown in Figure 11, the tannin, lignin, TOC, TP contents had significant impacts on the bacterial community, with the order of influence of the primary factors explaining total bacterial community variation being tannins > lignin > TOC > TP > TN > cellulose. For *Canna glauca* leaf litter, the first and second principal component axes accounted for 52.70% and 32.40% of the variation in the bacterial community, respectively. The chemical properties notably impacting the bacterial community were TOC, cellulose, TP, and tannin contents, with their order of influence being TOC > cellulose > TP > tannin > lignin > TN.

## 3. Discussion

### 3.1. Tannins Exhibit Non-Linear Regulatory Effects on Leaf Litter Decomposition Rates

This study revealed that tannins exhibit a non-linear effect of “low-concentration promotion and high-concentration inhibition” during leaf litter decomposition. This phenomenon is not only closely related to the chemical properties of tannins and their dose-dependent relationship with microbial activity but also provides typical empirical support for the stoichiometric imbalance theory of litter decomposition [37]. Previous studies have found that secondary metabolites (e.g., tannins), as key indicators of litter quality, can interact with primary nutrient elements (e.g., nitrogen) [17], jointly regulating the decomposition rate. In the low-tannin treatment group (ABC1), the increase in decomposition rate is attributed to two factors: the high nitrogen content and low C/N ratio of *Myriophyllum verticillatum*, and the degradation of low-concentration tannins by microbes into small molecules that can be utilized metabolically [7,23]. Compared to the AB group, the decomposition rates of *Osmanthus* and pink canna in ABC1 increased by 1.33% and 12.71%, respectively. Furthermore, the cumulative mass loss rate of the ABC1 group was significantly higher than that of the AB control group at the end of the experiment (*p* < 0.05). This promotive effect aligns with the concept of the priming effect [38]—a phenomenon widely reported in both aquatic and terrestrial ecosystems. Specifically, the input of labile organic compounds (i.e., small-molecule intermediates from low-concentration tannin degradation in the ABC1 group) stimulated microbial metabolic activity, which in turn accelerated the decomposition of recalcitrant organic matter in leaf litter of *Osmanthus fragrans* and *Canna glauca*. Partial degradation products of tannins (e.g., gallic acid) can serve as readily available carbon sources [19], promoting the proliferation of specific microbial taxa such as *Bacteroidetes*. Additionally, low concentrations of phenolic compounds can induce microorganisms to produce antioxidant enzymes (e.g., catalase), effectively scavenging reactive oxygen species (ROS) generated during decomposition, thus protecting intracellular enzyme systems (e.g., cellulase, laccase) from oxidative inactivation [39]. This low-dose stimulatory effect (Hormesis) has been widely reported in plant-microbe interactions [40]. The establishment of this antioxidant system can prolong the metabolically active period of functional decomposer microbes [41], thereby indirectly enhancing the decomposition capacity of leaf litter.

However, in the high-tannin treatment group (ABC3), when the tannin concentration exceeded the ecological threshold, its toxic inhibitory effect became dominant. Specifically, the degradation rates of *Osmanthus* and pink canna litter in the ABC3 group decreased by 6.21% and 6.82%, respectively, compared to the AB group. This aligns with predictions from the substrate-microbe interaction model [42], which posits that high concentrations of secondary metabolites can negatively feedback on the decomposition process by altering substrate availability and exerting direct biotoxicity. Kinetic analysis in this study further revealed the dose-dependent nature of this inhibition: the decomposition rate constants (*k*-values) for both litter types in the ABC3 group were significantly lower than those in the AB and ABC1 groups (*p* < 0.01). High tannin concentrations primarily inhibit organic matter decomposition through the following pathways: (1) Enzyme Activity Inhibition: Tannins can bind to hydrolytic enzymes (e.g., cellulase, lignin peroxidase) via hydrogen bonding or hydrophobic interactions [43], reducing their catalytic efficiency. For instance, the complexation of tannins with cellulase directly impedes the enzymatic cleavage of cellulose chains [44]. Previous studies have found that microbial extracellular enzyme activity decreases exponentially with increasing polyphenol concentration [45]. (2) Substrate Masking Effect: Tannins form insoluble complexes with substrates in the litter, such as proteins and polysaccharides (e.g., tannin–protein precipitates) [46], thereby reducing the available carbon and nitrogen sources for microbes. (3) Microbial Toxicity: The phenolic hydroxyl groups of tannins can disrupt the membrane integrity of microbial cells [47], consequently inhibiting their growth and metabolic activity. This mechanism is consistent with the classical assertion that high tannin concentrations inhibit the growth of various microorganisms [48]. In this study, the high-tannin environment in the ABC3 treatment group led to a significant reduction in the alpha diversity index of the bacterial community during the later stages of litter degradation, with sensitive bacterial taxa being selectively suppressed, providing strong support for this mechanism. In summary, the chemical inhibitory effects of tannins can offset the inherent nutrient-promoting effects of high-quality litter, highlighting the precedence of chemical defense substances in regulating decomposition.

### 3.2. Inhibitory Effect of Tannin–Nitrogen Complexation on Leaf Litter Decomposition

The accumulation of TN in the leaf litter within the high-tannin treatment group (ABC3) was closely linked to tannin-nitrogen complexation. A significant nitrogen accumulation was observed in the leaf litter of *Osmanthus fragrans* in the ABC3 group. By the conclusion of the 140-day experiment, the TN content in the leaf litter across the three tannin-amended treatments followed the order: ABC3 > ABC2 > ABC1. Specifically, compared to the AB group, the TN content in the leaf litter of *Osmanthus fragrans* and *Canna glauca* was 26.46% and 17.69% higher, respectively. The addition of high levels of tannin inhibits nitrogen release, leading to its accumulation [49]. Tannins form stable complexes with proteins via their phenolic hydroxyl groups [46], which reduces nitrogen mineralization and promotes nitrogen immobilization. This mechanism is a central feature of the “nitrogen immobilization” phase during decomposition [50]. This study further reveals that the observed suppression of decomposition under high-tannin conditions may stem from what can be termed tannin-mediated “ineffective nitrogen immobilization” [19], wherein the immobilized nitrogen remains largely inaccessible for metabolic use by decomposer microorganisms. Consequently, this deprivation of bioavailable nitrogen likely constrains the microbial synthesis of nitrogen-rich lignin-degrading enzymes (e.g., lignin peroxidase, laccase) [13,17]. This enzymatic inhibition, coupled with the direct retardation of recalcitrant organic matter degradation by stable tannin-nitrogen complexes [21], collectively contributes to the overall slowing of litter decomposition.

During leaf litter degradation, the NAI-N dynamics result from the combined effects of the changing TN concentration in the remaining litter and the declining litter mass. The increase in TN concentration in the residual litter is partially counteracted by the reduction in biomass, leading to an eventual decline in the NAI-N value. Across various degradation stages, the NAI-N values in the three tannin-treated groups predominantly followed the trend ABC3 > ABC2 > ABC1, indicating that the ABC3 treatment exerted the most pronounced effect on nitrogen accumulation during degradation. The initial rise in TN content observed in *Osmanthus fragrans* litter during early degradation stages is likely attributable to its initially low TN content, facilitating microbial-mediated net nitrogen immobilization from the external environment into the plant residues [51]. In summary, tannins not only regulate the litter degradation rate but also, by influencing this process, indirectly impact aquatic nitrogen levels and the nitrogen cycle at the plant-water interface.

### 3.3. Tannin-Driven Dynamics of Aquatic Nitrogen

Tannins regulate the release of nutrients from decomposing leaf litter, influencing the aquatic nitrogen cycle. Our findings indicate that nutrients are rapidly released during the initial stages of leaf litter degradation. Within the first 14 days of litter degradation, the TN and tannin contents in the aquatic environment increased sharply. Hydrolyzable tannins dissolved quickly [35], and the TN content in the water reached its highest level of 29.92 mg/L in the ABC1 group. During this phase, the soluble organic carbon from the leaf litter dissolved rapidly, and under sufficient dissolved oxygen in the aquatic environment, organic nitrogen was oxidized into inorganic nitrogen. As a result, the TN content in all the treatment groups peaked within a short period. The leaching and mineralization of organic nitrogen also led to a significant increase in the NH_4_^+^-N concentration [52]. After 14 days of degradation, the TN and tannin contents in the water began to decrease. During the degradation process, a biofilm capable of adsorbing nitrogen formed on the walls of the experimental containers. Additionally, nitrification and denitrification processes occurring in the water [53] converted inorganic nitrogen into nitrous oxide, which was eventually released as nitrogen gas [54].

During the mid-degradation phase, the tannin and TN contents in the water of all the treatment groups remained relatively stable but significantly decreased in the later stages. The trends in the TN and NH_4_^+^-N contents in the water across all treatment groups were similar and were characterized by an initial increase followed by a decrease. As the NH_4_^+^-N content in the aquatic environment decreased, the NO_3_^−^-N concentration gradually increased, indicating the conversion of NH_4_^+^-N into NO_3_^−^-N under the action of nitrifying bacteria [55]. Compared to the typical freshwater nitrogen cycle pattern, high tannin input (ABC3 group) significantly altered the transformation dynamics and proportions of various nitrogen forms, leading to a relative accumulation of NH_4_^+^-N. This phenomenon is likely associated with the inhibitory effect of tannins on nitrifying bacteria [56]. A comparison of changes in the NH_4_^+^-N content among the different degradation groups revealed that the NH_4_^+^-N levels fluctuated during the mid-to-late degradation stages. In the later stages, the NH_4_^+^-N contents in treatment groups A, AB, ABC2, and ABC3 increased, and the impacts of the NH_4_^+^-N contents in the litter in these groups on aquatic NH_4_^+^-N were significantly greater than those in treatment groups B and ABC1. At the end of the experiment, the NH_4_^+^-N content in the water of the ABC3 group was 59.29% greater than that of the AB group, whereas the NH_4_^+^-N content in the water of the ABC1 group was 17.70% lower than that of the AB group.

### 3.4. Tannin-Mediated Regulation of Bacterial Community Succession

Our study revealed that the succession of bacterial communities during leaf litter degradation is closely related to tannin content. Bacteria contributed to decomposition throughout the entire process, but their functional roles exhibited distinct phased characteristics. This observation aligns with the “environmental filtering” theory, which posits that environmental factors (e.g., tannins) exert selective pressure on microbial communities [57]. Bacteria are involved throughout the degradation process, but their functional roles are stage specific. In the later stages of degradation, tannin accumulation reshaped the microbial community structure through a dual inhibitory effect—nutrient limitation [51] and toxic stress [7]—promoting oligotrophic and stress-resistant bacterial species as dominant functional groups [58,59]. This drove the transformation of recalcitrant organic matter. At high concentrations [35], tannins significantly inhibit the release of nutrients such as proteins through chemical complexation, leading to an imbalance in the carbon–nitrogen ratio in the later stages of degradation and creating a “nutrient bottleneck”. Additionally, tannins with phenolic hydroxyl structures can penetrate microbial cell membranes, interfere with enzyme activity, and induce oxidative stress [35,58].

Our findings revealed that in the ABC3 treatment group, the bacterial α diversity indices were lower than those in the AB group during the later stages of degradation. Specifically, the Simpson indices for *Osmanthus fragrans* and *Canna glauca* leaf litters in the ABC3 group were 44.44% and 25.00% lower, respectively, than those in the AB group; similarly, the ACE index was 11.98% and 8.64% lower, respectively. These results validate the filtering effect of selective pressure due to tannin toxicity on the microbial community.

During the decomposition of leaf litter, the composition of organic matter continuously changes, leading to corresponding shifts in the structure and species abundance of microbial communities. The phyla *Bacteroidetes* and *Firmicutes* were significantly positively correlated with tannin content during the degradation of *Osmanthus fragrans* leaf litter. In the early stages of degradation, *Bacteroidetes* dominated the microbial community, but its abundance gradually decreased in the mid-to-late stages. *Bacteroidetes* thrive in relatively nutrient-rich environments. During the initial degradation phase, easily degradable nutrients were rapidly released, and the abundance of *Bacteroidetes* in the ABC1 group (80.42%) was significantly greater than that in the ABC3 group (53.71%). *Bacteroidetes* play a leading role in the utilization of readily decomposable carbon sources, and the efficient degradation of these sources accelerates the initial decomposition of leaf litter [60].

In the later stages of degradation in the ABC3 treatment group, the dominant bacterial genus in *Canna glauca* leaf litter was *Treponema*. *Treponema*, a Gram-negative anaerobic bacterium within the phylum *Spirochaetes* [61], primarily utilizes simple organic compounds such as proteins and short peptides [62]. During the later stages of litter degradation, the high tannin content in the ABC3 group inhibited cellulose- and lignin-degrading bacteria (e.g., *Fibrobacter*) [21]. In the high-tannin environment, many tannin-tolerant fungi—recognized as primary decomposers of complex organic compounds like lignocellulose [63]—were likely constrained (e.g., by reduced synthesis of lignocellulolytic enzymes) [17], limiting their ability to decompose recalcitrant substrates. Instead, *Treponema* became dominant: this bacterium acquires carbon and nitrogen sources by degrading free proteins or short peptides not bound by tannins [64], but it is not a primary decomposer of complex organics (e.g., cellulose or tannins). Moreover, *Treponema*’s low metabolic efficiency (e.g., reduced ATP yield) [65,66] further compounded the decomposition constraint—together, the suppression of fungal primary decomposers and dominance of low-efficiency *Treponema* led to a significant decline in overall decomposition rates [67]. This succession from a “eutrophic” microbial community to a “stress-tolerant” one directly reflects the functional degradation of decomposition under high-tannin conditions. This pattern is consistent with observations in terrestrial systems, where high phenolic compound levels lead to functional simplification of decomposer microbial communities [20].

### 3.5. Ecological Management Implications

The non-linear regulatory effect of tannins on litter decomposition revealed in this study provides new insights into global litter decomposition patterns. Plant tannin content decreases with increasing latitude, while the decomposition rate of riparian litter increases with latitude [8]. Additionally, litter in tropical-subtropical regions exhibits higher recalcitrance and slower decomposition rates [11]. The high-tannin inhibition effect identified in this study provides a mechanistic explanation for these low decomposition rates.

Based on the “low-promotion and high-inhibition” effect of tannins on decomposition, riparian zone plant configurations can be optimized as follows: In temperate regions, mixed planting of high-tannin tree species with fast-growing low-tannin species maintains decomposition rates. In tropical-subtropical regions, avoid excessive aggregation of high-tannin species (e.g., *Eucalyptus*) to reduce decomposition inhibition; instead, implement mixed planting of low-tannin plants (e.g., Salix) and introduce nitrogen-fixing trees (e.g., *Albizia*) to balance decomposition rates and nitrogen retention needs. Regular removal of high-tannin litter accumulation zones reduces inputs to aquatic systems.

Enhancing nitrogen availability alleviates inhibition from tannin-nitrogen complexation. Apply slow-release nitrogen fertilizers (e.g., coated urea) in litter-enriched areas with strict dosage control to prevent eutrophication. Protect nitrifying bacterial communities at aquatic-terrestrial interfaces (e.g., by maintaining aerobic microenvironments) to promote ammonium-to-nitrate conversion. Selectively retain high-tannin vegetation in agricultural stream riparian zones to utilize its nitrogen interception capacity for runoff purification and non-point source pollution mitigation. Note: While slow decomposition of high-tannin litter enhances short-term soil carbon storage, monitor risks of CH_4_ emissions under anaerobic conditions [8].

Directional cultivation of functional microbial communities and long-term monitoring with adaptive management are essential. Introduce efficient lignin-degrading bacteria [31] into riparian soils to enhance degradation of recalcitrant material. Conduct real-time monitoring of litter tannin/nitrogen stoichiometric ratios and aquatic ammonium/nitrate nitrogen dynamics. Dynamically adjust vegetation configurations and hydrological management based on monitoring data.

## 4. Materials and Methods

### 4.1. Plant Species

A survey was conducted to statistically analyze leaf litter from urban green spaces in Nanning city. On the basis of survey data and a comprehensive review of the relevant literature, representative and widely distributed aquatic plants from urban green spaces and terrestrial plants along water bodies were selected for this study. Leaf litter from these plants was collected, and their tannin content was precisely measured. Litter from two low-tannin plant species commonly found in Nanning’s urban green spaces was ultimately chosen as the decomposition substrate: litter from the terrestrial plant *Osmanthus fragrans* (A) and the aquatic plant *Canna glauca* (B). The high-tannin aquatic plant *Myriophyllum verticillatum* (C) was selected as an additive. Experimental groups with varying tannin concentrations were established to conduct decomposition experiments. The initial physicochemical properties of the three plant species are presented in Table 2. The tannin content was 130.02 mg/g in *Myriophyllum verticillatum* leaves, compared to 18.69 mg/g and 12.20 mg/g in the leaf litter of *Osmanthus fragrans* and *Canna glauca*.

### 4.2. Experimental Design

From December 2022 to mid-April 2023, fresh leaf litter or senescent leaves of *Osmanthus fragrans* and *Canna glauca* were collected from lakeside areas on the campus of Guangxi University. The collected leaf litter was rinsed with deionized water and air-dried in the laboratory for 2 days. The litter was subsequently placed in envelopes and dried in an oven at 65 °C until a constant weight was achieved. The experiment included two single-species litter treatments (treatment A: *Osmanthus fragrans*; and treatment B: *Canna glauca*), one mixed litter treatment (treatment AB: *Osmanthus fragrans* + *Canna glauca*), and three treatments with *Myriophyllum verticillatum* tannin added (treatments ABC1, ABC2, and ABC3). In this context, treatment group AB served as the control. It was designed to compare the degradation rates of leaf litter after the addition of different tannin gradients to the AB combination, and to contrast these rates with the degradation rates of Plant A and Plant B individually. Additionally, a separate degradation group for *Myriophyllum verticillatum* (Treatment C) was included, with each bag containing 10 g of litter. The initial dry weight of each treatment is detailed in Table 3. Litter from each plant species was individually placed into 10 cm × 10 cm decomposition bags with a mesh size of 100 mesh. For the mixed treatments, the litter was placed in 20 cm × 25 cm nylon mesh litter bags. A total of 200 bags were prepared (6 treatments × 3 replicates × 7 sampling events + spare bags). Polyethylene resin boxes with specifications of 120 L and 240 L were used as test chambers. Test water was prepared by mixing tap water with lake water from the campus of Guangxi University at a ratio of 99:1. (The addition of a small quantity of lake water (1%) was primarily intended to introduce microbial communities from the natural aquatic environment, thereby better simulating the actual ecological decomposition process.) Decomposition bags were placed in the experimental boxes, with the initial biomass density of litter set to 2.10 g/L for each treatment. The experiment was conducted under light-shielded conditions at a controlled temperature of 25 °C, starting on 25 April 2023, with a duration of 140 days.

### 4.3. Sample Collection and Analysis

After the start of the experiment, litter decomposition bags were collected using destructive sampling on days 7, 14, 21, 28, 49, 70, and 140, with 3 bags sampled per treatment each time. The leaves in the decomposition bags were removed and dried to constant weight in an 85 °C drying oven, then ashed at 500 °C for 4 h to determine the ash-free dry mass for calculating the litter residual rate.

The dried leaves were pulverized, ground thoroughly with an appropriate amount of liquid nitrogen, and passed through a 100-mesh sieve for chemical analysis. Tannins were extracted using a 70% acetone solution, and their content was determined within 48 h using a modified Folin–Ciocalteu method [68]. To minimize interference from non-tannin substances (e.g., reducing sugars, amino acids), the following targeted measures were implemented: (1) Selective extraction with 70% acetone solution was employed to reduce the dissolution of water-soluble interferents. (2) The extracts were purified via adsorption using polyvinylpolypyrrolidone (PVPP), with the PVPP-treated group serving as the background to subtract non-specific interference. (3) Tannic acid was used as the standard for quantitative analysis. (4) The time interval from extraction to determination was strictly controlled within 48 h to minimize the influence of sample degradation.

Total organic carbon (TOC) content was measured using a Total Organic Carbon analyzer (Analytik Jena Multi N/C3100; Analytik Jena AG, Jena, Germany). Samples were digested with sulfuric acid (H_2_SO_4_)-perchloric acid (HClO_4_) to prepare test solutions, and then total nitrogen (TN) and total phosphorus (TP) contents were determined using a SmartChem 200 automated discrete chemical analyzer (AMS, Rome, Italy). Cellulose and lignin contents were measured using the extraction method described by van Soest [69] with an ANKOM fiber analyzer (A2000I, ANKOM, New York, NY, USA). Three parallel determinations were performed for each index.

Water samples were collected on days 14, 21, 49, 70, and 140. Before each collection, the water in the experimental boxes was thoroughly stirred. After collection, the water samples were first placed in 500 mL polyethylene bottles, filtered through filter paper with a pore size of 12–20 μm, then transferred to 100 mL polyethylene bottles and stored in a 4 °C refrigerator. Tannin content was determined using the modified Folin–Ciocalteu method described above. TN, ammonium nitrogen (NH_4_^+^-N), and nitrate nitrogen (NO_3_^−^-N) contents in water were measured using a SmartChem 200 automated discrete chemical analyzer (AMS, Rome, Italy). All water quality indicators were determined in 3 parallels and completed within 24 h.

### 4.4. DNA Extraction, Sequencing, and Bacterial Analysis of Leaf Litter

Leaf litter samples for DNA extraction were collected on days 30, 70, and 140 of the incubation. During each sampling event, 3 g samples were weighed from each litter type within every treatment group, placed into sterile bags, sealed, labeled, and stored at −80 °C in an ultra-low-temperature freezer for subsequent molecular biological analysis.

Microbial DNA was extracted using the FastDNA^®^ Spin Kit for Soil (MP Biomedicals, Santa Ana, CA, USA), and its quality was checked via NanoDrop 2000 (Thermo Fisher Scientific, Waltham, MA, USA) and agarose gel electrophoresis. High-throughput sequencing of the V4 region of the 16S rRNA gene was outsourced to Majorbio (Guangdong, China), using the 515F/806R primer pair for amplification. Bacterial identification was achieved through bioinformatic analysis: quality control of sequencing data, OTU clustering at 97% similarity, and taxonomic annotation by comparison against the SILVA database.

Bacterial abundance was quantified via qPCR. A standard curve was generated using 10-fold serial dilutions of a pMD18-T plasmid (Takara Bio, Shiga, Japan) cloned with a fragment of the 16S rRNA gene. The 20 μL reaction mixture contained 16.5 μL ChamQ SYBR Master Mix (Vazyme Biotech, Nanjing, Jiangsu, China), 0.8 μL each of the 515F and 806R primers, and 2 μL of DNA template. Reactions were performed on an Applied Biosystems 7500 instrument (Thermo Fisher Scientific, Waltham, MA, USA), and gene copy numbers were calculated based on the standard curve.

### 4.5. Data Processing and Analysis

All mass residual rates during decomposition of dying leaves were calculated using ash-free dry matter weight. The residual rate was calculated using the following formula [70]:

(1) $$Residual\;ratio=M_{t}/M_{0}\times 100\%$$

where $M_{0}$ represents the initial dry weight of the sample, and $M_{t}$ represents the dry weight of the sample at time *t* (days).

The theoretical predicted residual ratio ($K_{p}$) for mixed leaf litter was calculated as follows [21]:

(2) $$K_{p}=a\times K_{A}+b\times K_{B}+c\times K_{C}$$

where $K_{A}$, $K_{B}$, and $K_{C}$ represent the residual ratios of samples A, B, and C when decomposed individually, and *a*, *b*, and *c* represent their respective proportions in the mixed treatment groups.

The decomposition rate (*k*) of leaf litter was fitted using an exponential decay model [71]:

(3) $$M_{t}=M_{0}\times e^{-kt}$$

where $M_{t}$ is the residual mass of leaf litter after time *t* (days), $M_{0}$ is the initial dry weight of the leaf litter, *k* is the decomposition rate constant, and *t* is the decomposition time (days).

The nutrient accumulation index (NAI) during leaf litter decomposition was calculated as follows [72]:

(4) $$NAI=(M_{t}\times X_{t})/(M_{0}\times X_{0})\times 100\%$$

where $M_{t}$ is the dry mass at time *t*, $X_{t}$ is the element concentration (mg/g) at time *t*, $M_{0}$ is the initial dry mass, and $X_{0}$ is the initial element concentration (mg/g).

Statistical analyses were performed using SPSS 22.0 software. One-way ANOVA and Pearson correlation analysis were conducted to evaluate significant differences and relationships among variables. Data organization and figure preparation were performed using Excel 2023 and Origin 2023. All data are presented as means, and unless otherwise specified, significant differences were determined at the *p* < 0.05 level.

Operational taxonomic units (OTUs) were clustered at a 97% similarity threshold. The OTU table was used to calculate microbial α-diversity and analyze the relative abundance of bacterial communities at the phylum, class, and genus levels during the early, middle, and late stages of leaf litter decomposition. Correlation analysis between leaf litter physicochemical factors and microbial community structure, as well as visualization, was performed using cloud-based software platforms.

## 5. Conclusions

This study investigated the effects of adding tannin-rich plants at varying concentrations on the decomposition characteristics of mixed aquatic and terrestrial leaf litter under controlled temperature, shaded, and hydroponic conditions. The results demonstrate that tannins regulate the litter decomposition rate in a “low-concentration promotion, high-concentration inhibition” manner, a pattern primarily driven by the core mechanism of chemical complexation. This complexation directly perturbs the nitrogen cycle through nitrogen immobilization and restructures the microbial community by suppressing efficient decomposers. Collectively, these effects shift the organic matter turnover pattern from “rapid nutrient release” to “slow carbon decomposition.”

This study provides a new perspective and potential applications for interpreting differences in leaf litter decomposition patterns across global latitudinal gradients. Future research should focus on quantifying the relationship between the intensity of tannin complexation and its ecological effects and explore the use of tannin-rich plants in riparian zone management to synergistically enhance carbon sequestration and water quality.

## Figures and Tables

**Table 1:** Exponential equations and corresponding parameters for leaf litter residual rate (y) and decomposition time (*t*).

Specimen	R^2^	*K*	Formula	*t*_0.95_ (d)
A	0.911	0.003	y = 7.868e^−0.003t^	1521.19
B	0.991	0.009	y = 8.624e^−0.009t^	616.72
AB-A	0.909	0.003	y = 7.892e^−0.003t^	1670.42
AB-B	0.985	0.007	y = 8.126e^−0.007t^	741.95
ABC1-A	0.927	0.003	y = 7.919e^−0.003t^	1605.34
ABC1-B	0.980	0.008	y = 8.469e^−0.008t^	666.42
ABC2-A	0.938	0.003	y = 7.930e^−0.003t^	1730.53
ABC2-B	0.993	0.007	y = 8.272e^−0.007t^	741.68
ABC3-A	0.928	0.002	y = 7.881e^−0.002t^	2158.49
ABC3-B	0.968	0.007	y = 8.559e^−0.007t^	771.12

The exponential equation and its corresponding parameters between litter mass residual rate (y) and decomposition days (*t*) were fitted using an exponential decay model to calculate the litter decomposition rate (*k*) and determine the time required for 95% decomposition (i.e., *t*_0.95_) of litter in different treatment groups.

**Table 2:** Initial physicochemical properties of the selected plant species.

Specimen	Tannin	TOC	TN	TP	Cellulose	Lignin	C/N	C/P
	(mg/g)							
*Osmanthus fragrans*	18.69 ± 0.14 b	437.40 ± 6.9 a	12.51 ± 0.02 c	1.94 ± 0.6 c	235.06 ± 4.56 a	190.84 ± 2.61 a	34.96 ± 2.12 a	225.31 ± 3.64 a
*Canna glauca*	12.20 ± 0.11 c	381.90 ± 3.0 b	14.63 ± 0.07 b	2.22 ± 0.06 b	143.89 ± 2.89 b	112.01 ± 1.90 c	26.10 ± 0.08 b	172.24 ± 3.15 b
*Myriophyllum verticillatum*	130.02 ± 0.15 a	365.70 ± 2.2 c	25.45 ± 0.57 a	3.28 ± 0.04 a	155.28 ± 3.70 b	136.91 ± 2.67 b	14.37 ± 0.24 c	108.12 ± 2.02 c

Initial physicochemical properties of the three plant samples, including the contents of Tannin, TOC, TN, TP, Cellulose, Lignin, and the C/N and C/P values calculated from the contents. Values are means ± SE (*n* = 3). Different lowercase letters represent significant differences (*p* < 0.05).

**Table 3:** Initial dry weight of each treatment.

Treatment	Leaf Litter Species and Weight per Bag
A	*Osmanthus fragrans* (10 g)
B	*Canna glauca* (10 g)
AB	A (10 g) + B (10 g)
ABC1	A (10 g) + B (10 g) + C (3.85 g *Myriophyllum verticillatum*, containing 0.5 g tannins)
ABC2	A (10 g) + B (10 g) + C (19.23 g *Myriophyllum verticillatum*, containing 2.5 g tannins)
ABC3	A (10 g) + B (10 g) + C (34.61 g *Myriophyllum verticillatum*, containing 4.5 g tannins)

For the initial dry matter weight of each treatment, the following were set up: three single-species litter treatments (i.e., treatments A, B, and C); one two-species mixed litter treatment (i.e., treatment AB); and three tannin-added gradient treatments (i.e., treatments ABC1, ABC2, and ABC3). The amount of *Myriophyllum verticillatum* added was derived from calculations based on its tannin content.

## Data Availability

The original contributions presented in this study are included in the article/Supplementary Materials. Further inquiries can be directed to the corresponding author.

## Supplementary Materials

The following supporting information can be downloaded at: https://www.mdpi.com/article/10.3390/plants14193064/s1. Table S1: Bacterial diversity and richness of *Osmanthus fragrans* leaf litter at different decomposition stages. Table S2: Bacterial diversity and abundance during different decomposition periods of fading leaves of *Canna glauca*.
