# Supplementary material for: Regulatory Mechanisms of Tannins on the Decomposition Rate of Mixed Leaf Litter in Submerged Environments
Source: Plants (Basel). 2025 Oct 3;14(19):3064. doi: 10.3390/plants14193064 (PMC12526217; doi:10.3390/plants14193064)
Supplement: Supplementary file 1 [file plants-14-03064-s001.zip › plants-3859456-supplementary.pdf]

Supplementary Information

Table S1. Bacterial diversity and richness of *Osmanthus fragrans* leaf litter at different decomposition stages.

Sample Analysis Name	simpson	shannon_2	chao1	ACE	coverage
A1	0.28	3.63	864.50	1124.37	99.73%
AB1.A	0.31	3.44	881.20	1137.84	99.74%
L1.ABC1.A	0.61	2.03	717.30	947.00	99.80%
L1.ABC2.A	0.35	3.20	903.90	1211.29	99.73%
L1.ABC3.A	0.20	4.14	993.50	1251.45	99.72%
A2	0.09	5.26	1159.90	1438.01	99.65%
AB2.A	0.07	5.67	1191.00	1427.47	99.72%
L2.ABC1.A	0.05	5.94	1210.00	1448.61	99.72%
L2.ABC2.A	0.20	4.64	1141.70	1362.92	99.72%
L2.ABC3.A	0.13	4.96	1201.80	1417.81	99.71%
A3	0.15	5.08	1283.30	1639.51	99.69%
AB3.A	0.09	5.39	1334.80	1700.76	99.61%
L3.ABC1.A	0.10	5.85	1339.00	1708.49	99.71%
L3.ABC2.A	0.06	5.59	1224.00	1551.32	99.64%
L3.ABC3.A	0.05	5.21	1209.30	1496.94	99.67%

The α-diversity of bacterial community structure was reflected by Simpson, Shannon2, Chao1, ACE, and Coverage indices. The Simpson and Shannon2 indices indicate microbial community diversity, with higher values representing greater bacterial species diversity. The Chao1 and ACE indices reflect microbial richness, where higher values indicate higher bacterial richness and vice versa. The Coverage index is used to evaluate the representativeness of the detection results. As shown in the table, all Coverage indices exceeded 99%, indicating that the determined sequences could reflect the true status of the bacterial communities in the samples. (Note: A1, AB1.A, L1.ABC1.A, L1.ABC2.A, and L1.ABC3.A represent samples collected from treatments A, AB, ABC1, ABC2, and ABC3, respectively, for *Osmanthus fragrans* litter at 30 days of decomposition. The naming convention applies to other samples; the second and third sample collections were conducted at 70 and 140 days of decomposition, respectively.).

Table S2. Bacterial diversity and abundance during different decomposition periods of fading leaves of *Canna glauca.*

Sample Analysis Name	simpson	shannon_2	chao1	ACE	coverage
B1	0.05	5.65	1094.10	1376.78	99.62%
AB1.B	0.21	3.46	756.20	1022.81	99.74%
L1.ABC1.B	0.20	4.09	839.20	1199.29	99.66%
L1.ABC2.B	0.14	4.32	865.20	1088.68	99.73%
L1.ABC3.B	0.06	5.33	1066.00	1330.16	99.67%
B2	0.11	5.22	1110.50	1318.37	99.72%
AB2.B	0.14	4.31	820.80	993.51	99.79%
L2.ABC1.B	0.09	4.8	908.90	1087.70	99.78%
L2.ABC2.B	0.09	4.86	991.80	1203.12	99.73%
L2.ABC3.B	0.09	4.68	892.90	1089.69	99.76%
B3	0.10	5.48	1408.80	1744.99	99.65%
AB3.B	0.08	5.10	1190.20	1530.15	99.65%
L3.ABC1.B	0.11	5.18	1111.10	1441.08	99.67%
L3.ABC2.B	0.09	4.91	1109.50	1421.44	99.69%
L3.ABC3.B	0.06	4.69	1018.80	1398.00	99.69%

Bacterial diversity and richness of *Canna glauca* litter at 30, 70, and 140 days of decomposition. Herein, the Simpson and Shannon2 indices characterize the diversity of microbial communities, while the Chao1 and ACE indices characterize microbial richness.
